# KIAA1199 interacts with glycogen phosphorylase kinase β-subunit (PHKB) to promote glycogen breakdown and cancer cell survival

**DOI:** 10.18632/oncotarget.2220

**Published:** 2014-07-15

**Authors:** Masato Terashima, Yoshihiko Fujita, Yosuke Togashi, Kazuko Sakai, Marco A. De Velasco, Shuta Tomida, Kazuto Nishio

**Affiliations:** ^1^ Department of Genome Biology, Kinki University Faculty of Medicine, Osaka-Sayama, Osaka, Japan

**Keywords:** KIAA1199, glycogen phosphorylase kinase β-subunit, glycogen phosphorylase brain form, glycogen breakdown

## Abstract

The *KIAA1199* gene was first discovered to be associated with non-syndromic hearing loss. Recently, several reports have shown that the up-regulation of KIAA1199 is associated with cancer cell migration or invasion and a poor prognosis. These findings indicate that KIAA1199 may be a novel target for cancer therapy. Therefore, we explored in detail the function of KIAA1199 in cancer cells. In this study, we investigated the interaction of KIAA1199 protein with intracellular proteins in cancer cells. To this end, we expressed KIAA1199-MBP fusion protein and performed a pull-down assay. In addition, KIAA1199-overexpressing cancer cell lines were constructed using a retroviral vector and were used for further experiments. A pull-down analysis showed that the glycogen phosphorylase kinase β-subunit (PHKB) interacted with the C-terminal region of KIAA1199 protein. Furthermore, we observed the interaction of KIAA1199 with glycogen phosphorylase brain form (PYGB) under serum-free conditions. The interaction promoted glycogen breakdown and cancer cell survival. Our findings indicate that KIAA1199 plays an important role in glycogen breakdown and cancer cell survival and that it may represent a novel target for cancer therapy.

## INTRODUCTION

The *KIAA1199* gene is located on chromosome 15q25 and encodes a 150-kDa protein (1361 amino acids) that was first described as an inner ear protein in which three point mutations were found to be associated with non-syndromic hearing loss [1]. It has a G8 domain, containing eight conserved glycine residues and consisting of five β-strand pairs and one α-helix, four pbH1 domains, consisting of parallel β-helix repeats, and two GG domains, each consisting of seven β-strands and two α-helices. Recently, KIAA1199 was found to play a central role in hyaluronan binding and depolymerization [2].

Several reports have indicated that KIAA1199 is associated with cancer progression, metastasis and a poor prognosis. Specifically, the high expression of KIAA1199 in gastric tumors is associated with a poor prognosis and lymph node metastasis [3]. Moreover, the suppression of KIAA1199 attenuates Wnt-signaling and decreases the proliferation of colon cancer cells [4]. Other reports have indicated that the up-regulation of the *KIAA1199* gene is associated with the cellular mortality of normal human cells [5] and that KIAA1199 is a novel endoplasmic reticulum (ER) resident protein that plays a critical role in cancer cell migration and invasion through ER calcium release [6]. Also, KIAA1199 has been shown to play an important role in the growth and invasiveness of breast cancer cells [7].

These reports suggest that KIAA1199 contributes to cancer progression and may be a prospective target for cancer treatment. However, the putative cellular functions and pathway interactions have not been reported previously. We previously performed a microarray analysis of paired clinical samples of gastric cancer and noncancerous lesions obtained from gastric cancer patients [8] and found that KIAA1199 is overexpressed in gastric cancer tissue. The present study sought to clarify the biological function of KIAA1199 in cancer cell lines. To this end, we first constructed a maltose binding protein (MBP)-KIAA1199 fusion protein for use in a pull-down assay to identify proteins that specifically bind to KIAA1199 protein. In addition, cancer cell lines transfected with KIAA1199 cDNA were used to examine its biological behavior.

## RESULTS

### Tissue distribution of KIAA1199 mRNA in normal tissues and cell lines

**Figure 1:**
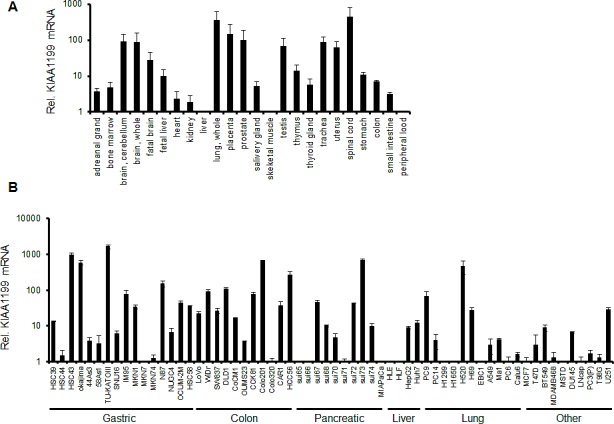
mRNA expression levels of KIAA1199 The mRNA expression levels were determined using a real-time RT-PCR analysis in (A) human normal tissues, (B) human cancer cell lines. GAPDH was used to normalize the expression levels. The data shown represent the average ± SD of three independent experiments.

To examine the tissue distribution of KIAA1199 mRNA, we performed real-time RT PCR using 24 normal human tissue samples. High expression levels of KIAA1199 mRNA were detected in the brain, placenta, and lung, whereas the levels in the liver, peripheral blood, bone marrow, and skeletal muscle were relatively low (Figure1A). These results were mostly consistent with a previous report describing the results of northern blotting [5], except that we additionally detected KIAA1199 mRNA in the prostate and spinal cord. KIAA1199 expression was also examined in 62 human cancer cell lines (Fig 1B). A relatively high mRNA expression level was observed in gastric cancer (TU-KATO III, okajima, and HSC43), colorectal cancer (Colo201 and COCM-1), pancreatic cancer (sui73), and lung cancer (H520). These results suggest that KIAA1199 is expressed in a variety of cancers especially those of digestive organs, such as stomach or colon (Figure 1B).

### Overexpression of KIAA1199 mRNA in gastric cancer tissues

**Figure 2:**
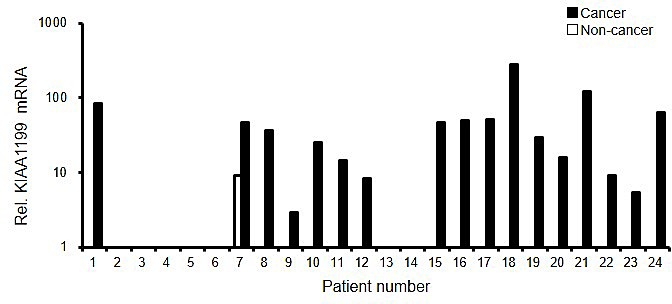
KIAA1199 mRNA was overexpressed in gastric cancer The mRNA expression levels were determined using a real-time RT-PCR analysis in paired tissues of gastric cancer and noncancerous gastric mucosa from 24 patients. GAPDH was used to normalize the expression levels.

The expression of KIAA1199 mRNA was analyzed for paired tissues of gastric cancer and noncancerous gastric mucosa obtained from 24 gastric cancer patients. The real-time RT PCR showed that KIAA1199 mRNA was dramatically overexpressed in gastric cancer tissues, compared with non-cancer tissues (Figure 2). These results are consistent with previous reports [3], in which six out of seven gastric cancer tissues expressed KIAA1199 at a level that was detectable using regular RT-PCR.

### Analysis of KIAA1199 binding proteins

**Figure 3:**
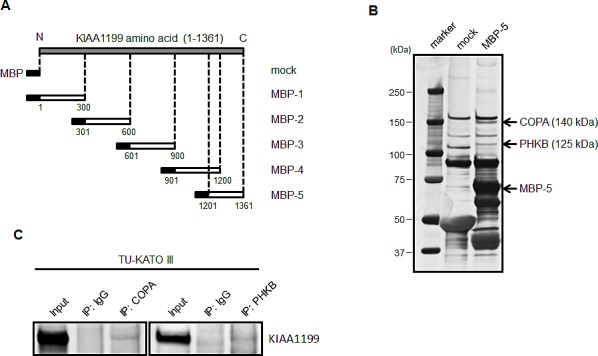
Construction of MBP-KIAA1199 fusion protein and detection KIAA1199 binding protein using a pull down assay (A) The schematic structure of five fragments of KIAA1199 used to fuse with MBP. The numbers represent the positions of the amino acids. All these fragments consisted of approximately 300 amino acids. (B) A pull down assay was performed using the fusion proteins with TU-KATO III whole cell lysate. The candidates were separated using SDS-PAGE, visualized using silver staining, and then analyzed using mass spectrometry. (C) An immunoprecipitation analysis was performed to confirm the interaction of COPA and PHKB with KIAA1199 in TU-KATO III cells.

To elucidate the biological function of KIAA1199, we constructed five variants of MBP-KIAA1199 fusion protein, as shown in Figure 3A, and forcibly expressed these variants in *E. coli*. Pull down assays were performed using purified recombinant proteins or MBP alone (mock) using a whole cell lysate of TU-KATOIII, a cell line expressing a high level of endogenous KIAA1199. The pull-down fraction of MBP-5, which corresponds to the C-terminal region of KIAA1199 protein, revealed two distinct protein bands, compared with the mock pull-down (Figure 3B). To identify the amino acid sequences of these putative KIAA1199 interacting proteins, the candidate bands were cut out from the gel and digested, and the resulting peptide fragments were analyzed using mass spectrometry. Peptide mass finger printing and tandem mass spectrum data from MALDI-TOF/MS were analyzed by searching against an MSDB or NCBI-nr database using MASCOT (Matrix Science, London, UK) search software. As described in Fig. 3B, these interacting proteins were identified as coatomer protein complex α-subunit (COPA, 140 kDa) and glycogen phosphorylase kinase β-subunit (PHKB, 125 kDa). The interactions of these proteins with KIAA1199 were confirmed using immunoprecipitation with the respective antibodies (Figure 3C).

### KIAA1199 enhanced the phosphorylation of glycogen phosphorylase brain form (PYGB)

**Figure 4:**
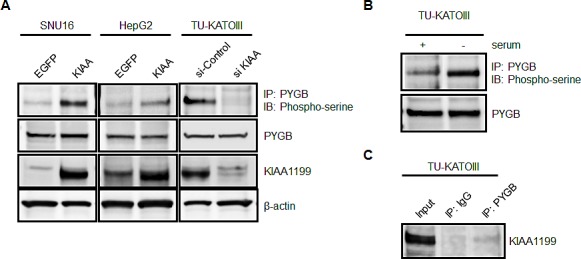
KIAA1199 enhanced the phosphorylation of PYGB in the absence of serum (A) To detect the phosphorylation of PYGB in SNU16, HepG2, and TU-KATOIII cells under serum-free conditions, immunoprecipitation obtained using anti-PYGB antibody was analyzed using western blotting with an antibody against phospho-serine residue. Western blotting analyses for PYGB, KIAA1199, and β-actin were also performed. (B) The phosphorylation of PYGB in TU-KATO III cells was enhanced under serum-free conditions. (C) The interaction of KIAA1199 with PYGB under serum-free conditions was observed in TU-KATO III cells using immunoprecipitation with a PYGB antibody followed by a KIAA1199 antibody.

Glycogen phosphorylase kinase (PHK) is a serine/threonine-specific protein kinase that phosphorylates a serine residue in glycogen phosphorylase (PYG), triggering the activation of this latter enzyme that catalyzes the phosphorolytic cleavage of the α-1,4 glycosidic linkages of glycogen, releasing glucose-1-phosphate as the reaction product. Since glycogen metabolism has recently been suggested to be a key pathway of metabolic reprogramming in cancer cells [9-12] and PHKB intracellularly binds KIAA1199, as shown in Figure 3, we questioned to what extent KIAA1199 is associated with glycogen breakdown. Several reports have demonstrated that glycogen phosphorylase brain form (PYGB) is overexpressed in various cancer tissues, including gastric cancer [13-15]. Based on this observation, we investigated whether the phosphorylation of endogenous PYGB occurs in SNU16, a cell line expressing a relatively low level of KIAA1199 (Figure 1B). As shown in Fig. 4A, the phosphorylation of PYGB was clearly observed when KIAA1199 was overexpressed in SNU16 (SNU16/KIAA1199), while it was barely detectable in the control cells (SNU16/EGFP). Similar results were obtained for the HepG2 cell line, a hepatocellular cancer cell line that moderately expresses KIAA1199 (Figures 4A and 1B). Interestingly, the phosphorylation of PYGB occurred in intact TU-KATO III cells and was enhanced by serum starvation (Figure 4B). This cell line expresses a relatively high level of KIAA1199, which may account for PYGB's susceptibility to phosphorylation. This hypothesis seems plausible because TU-KATOIII with KIAA1199 silenced by siRNA showed a marked reduction in the phosphorylation of PYGB (Figure 4A). Furthermore, immunoprecipitation showed a moderate interaction of KIAA1199 with PYGB under serum-free conditions (Figure 4C). These results suggest that KIAA1199 interacts with PYGB directly or indirectly through PHKB, resulting in the enhanced phosphorylation of PYGB under serum-free conditions.

### KIAA1199 promotes intracellular glycogen breakdown and cellular survival in the absence of serum

**Figure 5:**
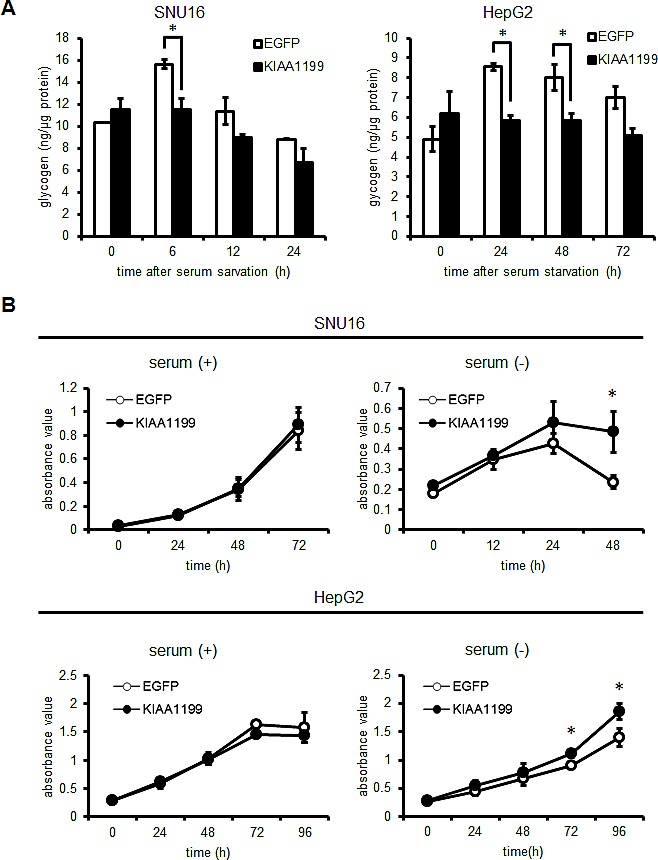
KIAA1199 promoted glycogen breakdown and extended cellular growth under serum-free conditions (A) Glycogen levels were measured in SNU16 cells and HepG2 cells in the presence or absence of serum. Open bars: EGFP-overexpressing cells (control); closed bars: KIAA1199-overexpressing cells. (B) The cellular growth of SNU16 and HepG2 cells was examined using an MTT assay in the presence (+) or absence (-) of serum. Open circles: EGFP-overexpressing cells; closed circles: KIAA1199-overexpressing cells. HepG2 cells in the presence of serum reached confluency at 72 h, causing an apparent decline in the growth rate. The data shown represent the average ± SD of three independent experiments. *: *P* < 0.05

To investigate whether KIAA1199 promotes glycogen breakdown by phosphorylating PYGB under serum-free conditions, cellular glycogen was measured after serum removal. As shown in Figure 5A, no significant difference between EGFP (control) and KIAA1199-overexpressing cells was seen in the presence of serum for both SNU16 and HepG2 cells; however, an accumulation of glycogen was observed in SNU16/EGFP cells (15.6 ± 0.4 ng/μg protein, serum starvation for 6 h) and HepG2/EGFP cells (8.5 ± 0.2, 8.0 ± 0.6 and 7.0 ± 0.6 ng/μg protein, serum starvation for 24, 48 and 72 h, respectively). These results were in contrast to the lower glycogen levels observed in SNU16/KIAA1199 cells (11.5 ± 1.0 ng/μg protein, for 6 h) and in HepG2/KIAA1199 cells (5.8 ± 0.3, 5.8 ± 0.4 and 5.1 ± 0.4 ng/μg protein for 24, 48 and 72 h, respectively). Next, we investigated the effect of serum starvation on cellular growth. As expected, no difference in cellular growth was seen between EGFP and KIAA119-overexpressing cells in the presence of serum. However, in the absence of serum, the cellular survival patterns differed between the two cell types (Figure 5B). While the SNU16/EGFP cell numbers peaked at 24 h and rapidly decreased at 48 h, the SNU16/KIAA1199 cells showed a significantly higher survival rate even after 24 h. The HepG2/KIAA1199 cells also grew significantly faster than the control cells after 72 to 96 h of serum starvation (Figure 5B). These results suggest that KIAA1199 mediates the breakdown of glycogen, resulting in prolonged cellular growth or survival under serum-free conditions. To explore if there is an additional factor that interacts with KIAA1199 in response to serum starvation, we also performed another pull down assay for serum-starved TU-KATOIII cells using recombinant proteins (MBPs-1 to -5 in Fig 3A), but no additional protein was detected (data not shown).

### KIAA1199 protected SNU16 cells from apoptosis induced by serum starvation

**Figure 6:**
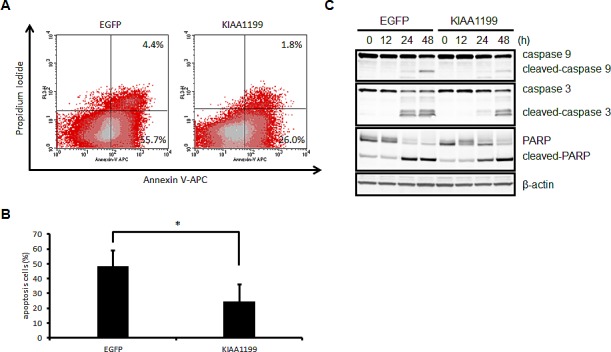
KIAA1199 prevented SNU16 cells from apoptosis induced by serum starvation (A) Flow cytometric analysis of SNU16-EGFP cells and SNU16-KIAA1199 cells after 24 h of serum starvation. Diagrams of Annexin V-APC/PI flow cytometry in a representative experiment are shown. (B) Apoptotic cells are expressed as the % of Annexin V-APC- plus PI-positive cells. The data shown represent the average ± SD of three independent experiments. *: *P* < 0.05 (C) Western blotting analysis for caspase-9, caspase-3, and PARP for their intact and cleaved forms. The numbers represent the time (h) after serum deprivation. β-actin was used as an internal control.

As shown in Fig. 5B, serum starvation inhibited SNU16 cell proliferation, probably by inducing apoptosis. We then investigated the effect of KIAA1199 overexpression on apoptosis. Cell apoptosis in the SNU16 cell line was estimated after 24 h of serum starvation using Annexin V staining and flow cytometry (Figure 6A). The apoptotic cell number was significantly decreased in SNU16/KIAA1199 cells (24.7% ± 11.3%), compared with SNU16/EGFP cells (48.2% ± 10.8%) (Figure 6B). A western blotting analysis also demonstrated that serum starvation induced the generation of cleaved caspase 3, cleaved caspase 9, and cleaved PARP, which are known apoptosis markers, in both SNU16/EGFP and SNU16/KIAA1199 cells, but the effects were delayed in the latter cell type (Figure 6C). These results indicated that KIAA1199 protects SNU16 cells from serum starvation-induced apoptosis through glycogen breakdown.

## DISCUSSION

The inner cell mass of a tumor is exposed to nutrient deprivation and hypoxia; in fact, glucose concentrations were much lower in tumor tissues than in normal tissues in both gastric cancer and colorectal cancer patients [16]. Energy deprivation can be a direct cause of cell death. Recent studies have suggested that glycogen synthesis is enhanced in both non-cancer and cancer cells when exposed to hypoxia, resulting in a large increase in glycogen accumulation that contributes to cell survival under oxygen- and glucose-free conditions [10-12]. In the present study, we also observed glycogen accumulation in control cancer cells under serum-free conditions. Thus, glycogen storage under nutrient-deprived conditions is an essential process for metabolic survival pathways in cancer cells. Concurrently, the process of glycogen breakdown is important for supplying glucose as energy for cell survival. Glycogen accumulation did not occur in KIAA1199-overexpressing cancer cells, while survival rate was relatively high in these cells, suggesting that KIAA1199 may accelerates glycogen breakdown, i.e., cancels glycogen accumulation, and eventually provides energy to cancer cells and thus prolonging proliferation by preventing cell death. In that case, the interaction observed between the C-terminal portion of KIAA1199 and PHKB, a subunit of an enzyme known to be related to glycogen breakdown, would be the key. An additional, yet unidentified allosteric factor that modulates the protein-protein interaction in response to serum starvation may be involved.

**Figure 7:**
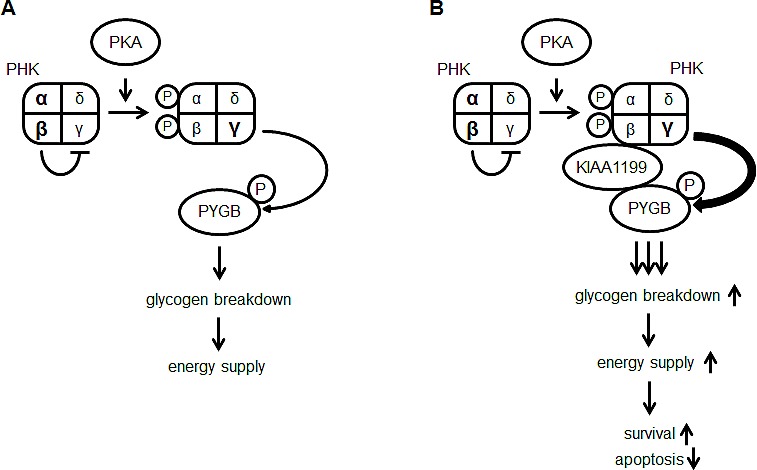
A proposed model of glycogen metabolism through the activation of PHK and PYG pathways in cancer cells with or without KIAA1199 overexpression (A) PKA phosphorylates the α- and β-subunits of PKH enhancing the γ-subunit activity and resulting in the phosphorylation of PYGB, leading to the activation of glycogen breakdown. (B) In KIAA1199-overexpressing cells, KIAA1199 interacts with PHK via the β-subunit and PYGB. This interaction facilitates PYGB phosphorylation by PHK, thereby promoting glycogen breakdown and cell survival.

PHK catalyzes the phosphorylation of glycogen PYG, thereby stimulating the breakdown of glycogen to form glucose 1-phosphate, which leads to energy production. PHK is an enzyme comprised of four subunits, of which the γ-subunit possesses enzymatic activity that is tightly regulated by the other three regulatory subunits in response to changes in intracellular Ca^2+^ concentrations (sensed by the δ-subunit, calmodulin) and cAMP levels that stimulate protein kinase A (PKA) to phosphorylate the α- and β-subunits (PHKA and PHKB) [17, 18]. Upon phosphorylation, these two subunits, which basically inhibit the activity of the γ-subunit, reduce their function as γ-inhibitors, resulting in the enzymatic activation of PHK (Figure 7A). In our study, when cells were exposed for short periods (10 min) with isoprenaline, a β1- and β2-adrenoreceptor agonist that activates PKA, the phosphorylation of PYGB was clearly increased in KIAA1199-overexpressing cells (Supplementary Figure 1). This response could be due to the enhanced phosphorylation of PHK by isoprenaline-activated PKA, which renders PYGB susceptible to phosphorylation in KIAA1199-overexpressing cells. Although the phosphorylation of PHKB in both control and KIAA1199-overexpressing cells could not be detected in our study because of the unavailability of a high-specificity antibody against PHKB, we speculated that the interaction of KIAA1199 with both PHKB and PYGB is strengthened under nutrient-deprived conditions and that the ternary interaction may locate PHK accessible to its substrate, PYG (Figure 7B).

PYG plays a central role in the mobilization of carbohydrate reserves in a wide variety of organs and tissues [19, 20]. There are three major isoforms for mammalian PYG, namely, isoforms of muscle (PYGM), liver (PYGL), and brain (PYGB); these isoforms can be distinguished by functional and structural properties as well as by the tissues in which they are predominantly expressed. Whereas the role of PYGB is poorly understood, it is generally thought to induce an emergency glucose supply by breaking down glycogen during a stressful period [20]. PYGB has been suggested to be the major isoform of PYG found in fetal tissues and tumor tissues [13-15]. In addition, a strong correlation was seen between the expression of PYGB and both gastric cancer and intestinal metaplasia, whereas no positive staining was observed for PYGB in normal gastric epithelial cells [21]. In a recent report, the depletion of PYGL resulted in irreversible glycogen accumulation that was associated with a reduced proliferation and a corresponding induction of senescence in cancer cell lines [22]. These results suggest that KIAA1199, as well as PHK or PYG, could be a molecular target for disrupting glycogen breakdown for cell proliferation.

We also identified COPA as a binding protein of MBP-5 (MBP-fused C-terminal region of KIAA1199 protein). COPA is α-subunit of the COP I complex and mediates a retrograde transport pathway that selectively recycles proteins from the cis-Golgi to the ER by binding to the di-lysine trafficking motif KKXX [23]. KIAA1199 protein consistently has a motif at the C-terminal sequence (KKKL), and a previous study has indicated that KIAA1199 is an ER resident protein. Hence, we hypothesized that KIAA1199 protein is transported to the ER by COPA. This hypothesis is also supported by the finding that smooth ER enables glycogen to be broken down to glucose [24].

In conclusion, we observed high expression levels of KIAA1199 mRNA in clinical gastric cancer tissue and demonstrated that KIAA1199 protein interacted with PHKB and PYGB in TU-KATO III cell lines. This interaction may induce the phosphorylation of PYGB and glycogen breakdown, resulting in cancer cell survival. These results provide novel insight into the biological function of KIAA1199 in cancer cells and suggest that glycogen breakdown may be a potential target for cancer treatment. Production of monoclonal antibodies against a C-terminal polypeptide of KIAA1199 and screening of the antibodies that prevent the protein-protein interaction between KIAA1199 and PHKB would be promising.

## MATERIALS AND METHODS

### Patients and samples

Paired samples (gastric cancer and noncancerous gastric mucosa) obtained from 24 patients were evaluated for KIAA1199 mRNA expression. This study was approved by the institutional review board of the National Cancer Center Hospital, and written informed consent was obtained from all the patients. Endoscopic biopsy samples were immediately placed in an RNA stabilization solution (Isogen, Nippongene, Tokyo, Japan) and stored at -80°C. Other biopsy samples obtained from the same location were reviewed by a pathologist to confirm the presence of tumor cells.

### Cell culture

Two human gastric cancer cell lines, SNU16 and TU-KATOIII, and a human hepatic cancer cell line, HepG2, were maintained in RPMI1640 medium (Sigma Aldrich, St. Louis, MO, USA) supplemented with 10% fetal bovine serum (FBS, GIBCO BRL, Grand Island, NY, USA). The cells were incubated in a humidified atmosphere of 5% CO_2_ and passaged every 3-4 days.

### Expression vector construction and viral production

The full-length cDNA encoding the human KIAA1199 gene was amplified using PCR with the following specific primers (KIAA1199-forward: 5′-CCGCTCGAGCGGGCCATGGGA GCTGCTGGGAGG-3′ and KIAA1199-reverse: ATAGTTTAGCGGCCGCATTCTTATTCA CAACTTCTTCTTCTTCAC). It was subcloned into a pCR-Blunt II-TOPO cloning vector (Invitrogen, Carlsbad, CA, USA) at the XhoI and NotI sites. The sequence of the PCR-amplified cDNA was confirmed using the Sanger dideoxy method. The cloned KIAA1199 cDNA was cut out of the pCR-Blunt II-TOPO cloning vector and transferred into a pQCLIN retroviral vector (Clontech, Palo Alto, CA, USA). The resultant construct includes a green fluorescent protein (GFP) gene incorporated downstream of an internal ribosome entry site sequence (IRES), enabling the direct monitoring of KIAA1199 expression in the transfected cells. The pQCLIN construct was co-transfected with a pVSV-G vector (Clontech) for the constitution of the viral envelope into the gpIRES-293 cells using FuGENE6 transfection reagent (Promega, Madison, WI, USA) according to the manufacturer's instructions. After 48 h of transfection, the culture medium was collected and the viral particles were concentrated by centrifugation at 10,000 rpm for 3 h at 4°C. The viral pellet was re-suspended in fresh medium and stored at -80°C. To obtain the stable SNU16 and HepG2 transfectants constitutively overexpressing KIAA1199, the viral vector was infected into each cell line. A viral vector harboring EGFP cDNA alone was used to establish the control transfectants. The stable viral transfectants overexpressing KIAA1199 and EGFP (control) in each cell line were designated as SNU16/KIAA1199 or HepG2/KIAA1166 and SNU16/EGFP or HepG2/EGFP, respectively. The methods used in this section have been described previously [25].

### Real-time reverse-transcription (RT) PCR

Total RNA was converted to cDNA using a GeneAmp^®^ RNA-PCR kit (Applied Biosystems, CA, USA). The cDNAs were then used for quantitative PCR analysis with SYBR^®^ Premix Ex Taq (TaKaRa, Otsu, Japan). Oligonucleotide primers specific for KIAA1199 (forward: 5′-CAGCTGGCTCACTCTGACCT-3′ and reverse: 5′-TCTTTAATGACCAGCTTGCC-3′) were purchased from Sigma Aldrich. The PCR was performed using Thermal Cycler Dice (TaKaRa) under the following conditions: 95°C for 5 min, 50 cycles of 95°C for 5 s, plus 60°C for 10 s. GAPDH was used as an internal control to normalize and compare each sample.

### siRNA targeting KIAA1199

KIAA1199 siRNA transfection was performed with the transfection reagent Lipofectamine RNAiMAX (Invitrogen) according to the manufacture's instruction. KIAA1199 siRNA oligonucleotides (forward: 5′- GGUAUUCAGCCGGAUCCUUTT-3′ and reverse: 5′-TTCCAUAAGUCGGCCUAGGAA-3′) were purchased from Sigma Aldrich.

### Cellular growth assay

Cell growth was assessed using a standard 3-(4, 5-dimenthyl-thiazoyl-2-yl) 2, 5-diphenyltet-razolium bromide (MTT) assay, which detects the dehydrogenase activity of viable cells. Briefly, 2000 cells/well were seeded in a 96-well plate and incubated for 24-72 h at 37°C. After incubation, 20 μL of MTT solution (Sigma Aldrich) was added to each well and the plates were incubated for 2 h at 37°C. The culture medium was removed, and the resultant MTT-formazan crystals were dissolved in 200 μL of DMSO per well. The absorbance was measured at 570 nm using VERSAmax (Molecular Devices, Tokyo, Japan). The experiment was performed in triplicate.

### Expression of MBP-KIAA1199 fusion proteins in *E. coli*

To express several MBP-KIAA1199 fusion proteins, we amplified cDNAs encoding 5 portions of KIAA1199, as shown in Fig. 3A, using the primers described in Table 1 and inserted the cDNAs into pMAL-c2 vectors (New England Biolabs, Ipswich, MA, USA). These vectors and a vector without insertion (mock) were used to transform *E. coli* (DH5α). Protein expression in the *E. coli* transformants was induced by the addition of isopropyl β-D-1-thiogalactopyranoside to a final concentration of 0.5 mM. When OD600 reached 0.8, the bacterial cells were harvested using centrifugation and the pellet was sonicated in column buffer containing 20 mM Tris-HCl, pH7.5, 200 mM NaCl, 1 mM EDTA, and a protease inhibitor cocktail tablet (Complete Mini; Roche Diagnostics, Basel, Switzerland). The bacterial cells were disrupted by adding 1% Triton X-100 and were centrifuged at 8,000 rpm for 10 min, and the resulting supernatant was applied to a maltose-binding protein affinity column (amylose resin, New England Biolabs) for purification. After washing with column buffer three times, the fusion protein was eluted from the column with a 10 mM maltose-containing column buffer. The elution fractions were examined using SDS-PAGE, and fractions containing the fusion protein were collected and dialyzed against replacement buffer (20mM Tris-HCl, pH7.5, 50 mM NaCl, 1 mM EDTA) overnight at 4°C. The dialysate was stored at −80°C.

**Table 1:** PCR primers used for cloning five portions of KIAA1199 cDNA

Primer name	Sequence
MBP-1F	5′-CGGAATTCCGGATGGGAGCTGCTGGGAGGCAGGACTTC-3′
MBP-1R	5′-CCCAAGCTTGGGTCAGAATACCCGGGCAGCAGCAGAGCCTCGATG-3′
MBP-2F	5′-CGGAATTCCGGAAATTGTTCCAGACAGAGCATGGCGAATAT-3′
MBP-2R	5′-CCCAAGCTTGGGTCAGATCAACAAGCCATTGGAGCCATGGACTGT-3′
MBP-3F	5′-CGCGGATCCGCGAAGGACGTTGTGGGCTATAAC-3′
MBP-3R	5′-CCCAAGCTTGGGTCAGCCCTCCAGGGCCACAAACTT-3′
MBP-4F	5′-CCGGAATTCCGCCGGCACACCAGCGCCCTGGCC-3′
MBP-4R	5′-CCCAAGCTTGGGTCAAAAGAGCTTCTTGGGCATCGG-3′
MBP-5F	5′-CCGGAATTCCGCCTGAAGCAAACGTCCAAGACG-3′
MBP-5R	5′-TGCTCTAGAGCATCACAACTTCTTCTTCTTCACCAC-3′

### Pull down assay

One milligram of TU-KATOIII whole cell lysate in column buffer was incubated with each of the KIAA1199-MBP fusion proteins at 4°C for 2 h. After incubation, 50% slurry amylose resin was added and the samples were further incubated for 1 h at 4°C. The amylose resin-bound fusion protein was washed with column buffer five times and eluted with the column buffer containing 10 mM maltose. The eluant was then separated using SDS-PGE and visualized using a silver stain kit (ATTO, Tokyo, Japan), according to the manufacturer's instructions.

### Western blotting

A western blotting analysis was performed as described previously [26]. Cells were washed twice with phosphate-buffered saline (PBS) and lysed by incubation in Lysis A buffer containing 1% Triton X-100, 20 mM Tris-HCl (pH7.0), 5 mM EDTA, 50 mM sodium chloride, 10 mM sodium pyrophosphate, 50 mM sodium fluoride, 1 mM sodium orthovanadate, a tablet of Complete Mini (Roche Diagnostics), and a phosphatase inhibitor cocktail (Sigma Aldrich). The proteins were resolved using SDS-PAGE and were transferred to a PVDF membrane (Immobilon; Millipore, Billerica, MA, USA). After blocking with Tris-buffered saline (TBS) containing 0.02% Tween 20 and 5% nonfat milk, the strips of membrane were exposed to anti-KIAA1199 antibody (epitope sequence, IHSDRFDTYRSKKESERLV; ordered from IBL, Fujioka, Japan), anti-phospho-serine antibody (Millipore), or anti-β-actin antibody (Cell Signaling, Beverly, MA, USA). The cells were incubated with HRP-conjugated anti-rabbit IgG antibody, and the proteins were visualized using an ECL Western Blotting Detection System (GE Healthcare, Buckinghamshire, UK).

### Immunoprecipitation

Cells were isolated and lysed in Lysis buffer A. The protein extract (500 μg) was incubated with anti-PHKB antibody (Bioss, Woburn, MA, USA), anti-PYGB antibody (Santa Cruz, Dallas, TX, USA), or anti-COPA antibody (Abcam, Cambridge, UK) overnight at 4°C. Protein G-agarose beads (Santa Cruz) were then added and allowed to bind to the complex for 90 min at 4°C. After the beads were washed with Lysis buffer A five times, the immunoprecipitated proteins were resolved using SDS-PAGE and were visualized using western blotting, as mentioned above.

### Measurement of intracellular glycogen

An intracellular amount of glycogen was measured using a glycogen assay kit (Biovision, Milpitas, CA, USA), according to the manufacturer's instructions. Briefly, 1 × 10^6^ cells were seeded in 6-cm dishes and incubated at 37°C. The cells were homogenized with dH_2_O on ice and heated for 5 min at 95°C. After 5 min of centrifugation at 13,000 rpm, the supernatants were measured for absorbance at 570 nm. The glycogen amount was normalized according to the protein concentration. The experiment was performed in triplicate.

### Apoptosis analysis

SNU16/EGFP and SNU16/KIAA1199 cells were harvested after 24 h of incubation under serum-free conditions and washed with PBS. The cells were stained with Annexin V-APC and propidium iodide (eBioscience, San Diego, CA, USA) according to the manufacturer's instructions, then analyzed for fluorescence using a flow cytometer (FACSCalibur, BD, Franklin Lakes, NJ, USA) and processed using Cell Quest software (BD).

### Statistical analysis

The statistical analyses were performed using Microsoft Excel (Microsoft) to calculate the SD and to test for statistically significant differences between the samples using the Student *t*-test. A *P* value of <0.05 was considered statistically significant.

## SUPPLEMENTARY MATERIAL FIGURE



## List of abbreviations

COPA: coatomer protein complex α-subunit
ER: endoplasmic reticulum
PHKB: glycogen phosphorylase kinase b-subunit
PYGB: glycogen phosphorylase brain form
RT: reverse-transcription
