# Supplementary material for: KIAA1199 interacts with glycogen phosphorylase kinase β-subunit (PHKB) to promote glycogen breakdown and cancer cell survival
Source: Oncotarget. 2014 Jul 15;5(16):7040–50. doi: 10.18632/oncotarget.2220 (PMC4196182; doi:10.18632/oncotarget.2220)
Supplement: Supplementary file 1 [file oncotarget-05-7040-s001.pdf]

KIAA1199 interacts with glycogen phosphorylase kinase β-subunit (PHKB) to promote glycogen breakdown and cancer cell survival

Supplementary Material

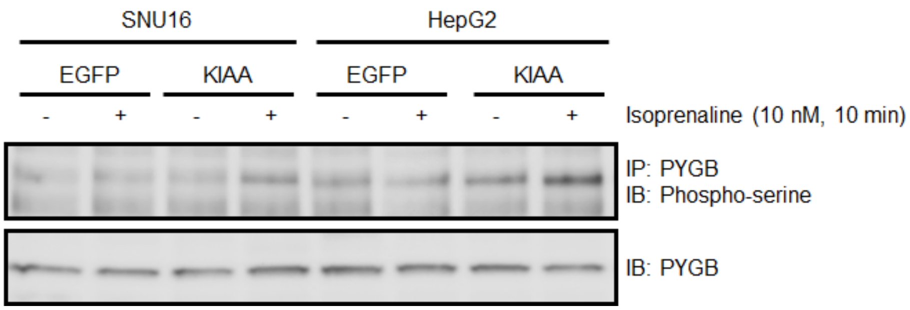

Supplementary Figure 1: KIAA1199 enhanced the phosphorylation of PYGB after exposure to isoprenaline (10 nM, 10 min) under serum-free conditions. Immunoprecipitation obtained using anti-PYGB antibody was analyzed using western blotting with an antibody against phospho-serine residue. Western blotting analyses for PYGB were also performed.
